# Caribou in the cross-fire? Considering terrestrial lichen forage in the face of mountain pine beetle (*Dendroctonus ponderosae*) expansion

**DOI:** 10.1371/journal.pone.0232248

**Published:** 2020-04-30

**Authors:** Barry R. Nobert, Terrence A. Larsen, Karine E. Pigeon, Laura Finnegan

**Affiliations:** 1 Caribou Program, fRI Research, Hinton, Alberta, Canada; 2 Alberta Environment and Parks, Grande Prairie, Alberta, Canada; 3 Grizzly Bear Program, fRI Research, Hinton, Alberta, Canada; 4 Geomatics and Landscape Ecology Research Lab, Carleton University, Ottawa, Ontario, Canada; University of British Columbia, CANADA

## Abstract

Mountain pine beetle (MPB) has become an invasive forest pest of mature pine in western North America as it spreads beyond its former endemic range. Management actions such as timber harvest can reduce the spread of MPB but may affect species of conservation concern like woodland caribou. Our goal was to inform MPB management within caribou ranges by exploring the impacts of MPB on caribou habitat–focusing on terrestrial lichens, an important winter food for caribou. We evaluated differences in lichen cover among four MPB management actions: timber harvest, wildfires, leaving MPB killed trees as-is, and single-tree cut-and-burn control. We found little evidence that leaving MPB killed trees as-is or controlling MPB using single-tree cut-and-burn impacted lichen cover. However, we found that lichen cover was lower in timber harvested and burned areas compared to intact undisturbed forest but only 10 to 20 years post-disturbance, respectively. Our results suggest that despite short-term reductions in lichen cover, using timber harvesting and prescribed burns to control MPB may balance management needs for MPB while maintaining lichen cover over time. However, using timber harvesting and prescribed burns to manage MPB is likely to have detrimental population-level effects on caribou by increasing the proportion of disturbed habitat and thus predators within caribou ranges. Among the four management actions that we evaluated, the cut-and-burn control program balances the need to limit the spread of MPB while also limiting negative impacts on caribou food. Our work addresses some of the challenges of managing competing forest and ecosystem values by evaluating the consequence of forest pest management actions on an important food resource for a species-at-risk.

## Introduction

Invasive species are a major source of ecological and economic loss [1–3]. In an effort to mitigate negative impacts of invasive species, land managers typically employ aggressive eradication programs [4,5]. However, management actions for species eradication can have unintended and detrimental ecological consequences on non-target organisms [6,7]. Because of this risk, managers should evaluate the potential for unintended outcomes prior to any intervention, especially in areas where management actions for invasive species could negatively impact species of conservation concern [8,9]. In such areas, evaluating the consequences of management actions on non-target species under alternate management scenarios could allow for proactive and informed invasive species mitigation [10,11].

Since the early 1990’s, land managers in western North America have attempted to control the spread of mountain pine beetle (*Dendroctonus ponderosae*; hereafter MPB); an endemic forest pest of mature pine west of the Rocky Mountains [12]. MPB breached the Rocky Mountains in the mid-2000s, spreading north and east at approximately 80km/year [13]. MPB has also spread from lodgepole (*Pinus contorta*) into jack pine (*Pinus banksiana*) [14,15] and whitebark pine (*Pinus albicaulis*) [16]. The spread of MPB into new regions, including western Alberta, is thought to be a result of decades of fire suppression associated with forest management coupled with increasingly warm winters and summers [15]. MPB is responsible for the death of millions of hectares of forest in Canada and the United States [17,18], with cascading and significant impacts on ecosystem function [19–21]. In an effort to eradicate and slow the spread of MPB, and to mitigate the economic impact on the forest industry, MPB control has focused on accelerated harvest of mature pine [22], single-tree cut-and-burn control programs of infested trees [23,24], and prescribed burning in protected areas [25]. However, MPB infestation and associated management actions can affect non-target organisms; a process that has already been observed in the boreal forest [26].

MPB and MPB management alter the forest structure by decreasing canopy cover and creating canopy gaps [27], which impacts understory vegetation by increasing light penetration and reducing snow interception [26,28,29]. For some boreal species, these impacts may be beneficial since species that prefer early seral habitats may benefit from changes in understory vegetation resulting in more food resources, and cavity-nesting birds may benefit from an increase in standing dead trees [26,29,30]. However, not all species benefit from MPB. For instance, elk (*Cervus canadensis*) avoid beetle-killed stands despite having abundant early seral forage, likely because of the increased costs of locomotion necessary to move through MPB-killed stands with dead and downed trees [31]. For species that prefer mature forest, MPB and MPB management may have significant and detrimental impacts on the availability of habitat and food resources [26,32]. For such species, specifically species that are of conservation concern, there is a need to evaluate whether MPB infestations could be less detrimental for species-at-risk compared to a range of MPB management actions.

Woodland caribou (*Rangifer tarandus*; hereafter ‘caribou’*)* are a threatened species [33–35] that prefer mature forest stands [36,37], which are habitats susceptible to MPB (e.g., mature pine). This preference for mature forest is driven by multiple factors but in part mature, open conifer stands provide abundant terrestrial and arboreal lichen that are important food for caribou during winter [38–40]. In addition, mature forest with sparse understory vegetation supports low densities of other ungulates and consequently, predators occur at low densities–effectively making contiguous mature forest stands predator refugia for caribou [41–43]. Loss and fragmentation of mature forest caused by habitat disturbances resulting in unstainable high predation rates is the main driver of caribou declines [44–46]. Because of this, federal and provincial caribou recovery strategies aim to reduce habitat disturbances such as timber harvest and wildfires within caribou ranges [34,35,47]. Because caribou conservation plans resolve to protect mature forest, they directly contradict the management actions for MPB eradication.

Management actions used to eradicate and slow the spread of MPB mainly accelerate harvest of mature pine in combination with single-tree cut-and-burn of infested trees at the leading edge of MPB spread [22,23,48]. In addition, stands that have already been killed by MPB may be salvage logged or burned [49] with prescribed burns being the main MPB management approach used in protected areas like National Parks [25]. These management actions are likely detrimental to caribou, but allowing MPB infestations to linger in caribou range may not guarantee the protection of caribou habitat either. For example, in British Columbia, Cichowski and Haeussler [50] reported a 9% decrease in percent cover of terrestrial lichens a decade after MPB infestation. These opposing management actions operating in the same region create a need to understand their potential impact to caribou habitat.

The goal of our study was to determine how MPB and alternative MPB management actions affect the distribution and abundance of terrestrial lichen in western Alberta. We focused on the impacts of MPB and MPB management on terrestrial lichen (hereafter “lichen”) because adequate food resources and nutrition are necessary to maintain sustainable caribou populations [51,52], and because caribou habitat use can be closely linked to the availability of forage [53,54]. First, we determined how MPB and actions to manage MPB affect lichen cover by constructing spatiotemporal lichen cover models. Specifically, we 1) modeled lichen cover in a) timber harvested stands, b) stands burned by wildfire, c) MPB single-tree cut-and-burn control stands, d) MPB infested stands, and e) intact stands. We then 2) simulated future lichen cover under different management actions. Second, we used resource selection functions (RSF; [55]) to evaluate the predictive ability of the lichen cover models based on the presumption that caribou should select habitats with higher predicted lichen cover. The results of this research are intended to help guide MPB management actions in support of caribou recovery in the boreal forest.

## Material and methods

### Ethical statement

Weyerhaeuser Company provided caribou GPS collar data with animal care protocols completed by Alberta Environment and Parks (AEP). AEP adhered to capture and handling guidelines under the Canadian Council on Animal Care [56] and the Government of Alberta’s Animal Care Protocol No. 008 [57]. Lichen data collection occurred on public lands and in provincial parks, and permission for field sampling and helicopter access was granted under the authority of the Government of Alberta (permit #14–109).

### Study area

The study area was approximately 33,000 km^2^, encompassed nine natural sub-regions [58], and included caribou ranges in west-central (53.857, -119.109) and north-western Alberta, Canada (57.675, -119.037). In west-central, forests are a mosaic of lodgepole pine, white spruce, and aspen, with black spruce, larch, and muskeg in low-lying areas [58–60]. Higher elevations have Engelmann spruce and subalpine fir below tree-line and graminoid, sedge, and herbaceous ground cover or exposed rock above tree-line. In the north-west, forests are white spruce, trembling aspen, and balsam poplar with black spruce, larch, and muskeg and fen in low-lying areas [58,61]. The study area included 2,032 km^2^ of federally protected land, 5,410 km^2^ of provincially protected land, and 25,761 km^2^ of provincial land-base. Hunting and other recreational activities occurred within protected lands but mostly on the provincial land-base. Industrial activities associated with the energy (mining, oil, and natural gas) and forest industry occur exclusively within the provincial land-base.

### Lichen absence and abundance

#### Field data collection

We used a geographic information system (GIS) and a random number generator to identify transects within forests stratified into five categories: timber harvest (*Cut*), wildfire (*Fire*), MPB kill (*MPB*), single-tree cut-and-burn control program (*SingleTree*), and intact undisturbed forest (*Forest*)–see S1 Appendix for details. For ease of access, we constrained 80% of transects to within 1 km of roads or pipelines and accessed the remainder via helicopter. We did not survey *SingleTree* within the north-western study area because at the time of data collection (2014 and 2015), there was no single-tree cut-and-burn management in the area. We collected data from 776 transects between June and October of 2014 and 2015 (S1 Appendix, Table 1).

**Table 1:** Transects surveyed among sampling strata.

Sampling strata	Description	Range (age in years)	West-central	North-western
Cut^a^	Regenerating timber harvest	1–49	193	65
Fire^b^	Regenerating natural wildfires	3–71	24	61
MPB^c^	Standing dead MPB killed pine trees	1–8	61	33
SingleTree^c^	Single-tree cut-and-burn control area	1–8	133	0
Forest	No history of timber harvest or natural disturbance	54–404	156	50

Number of transects surveyed in west-central and north-western Alberta, Canada among the five sampling strata during the summers of 2014 and 2015.

^a^ partitioned into time-since-disturbance in five year increments.

^b^ partitioned into time-since-disturbance in 10 year increments.

^c^ partitioned into time-since control or infestation in one year increments.

#### Field surveys and field-derived explanatory variables

We focused field surveys on four terrestrial lichen genera (*Cetraria* spp., *Cladina* spp., *Cladonia* spp., and *Flavocetraria* spp.) preferred by caribou [62]. At each transect, we visually estimated percent cover of lichens within six subplots placed at 5-m increments along a 25-m transect line. Because forest canopy cover and over-story species are known to influence the distribution and percent cover of lichen [63,64], we also estimated percent canopy cover at each subplot and recorded characteristics of the three nearest trees (species, MPB killed, and single-tree cut-and-burn control). Field data collection described in detail in S1 Appendix and field variables are in S2 Appendix.

#### GIS-derived explanatory variables

We linked transects to GIS-derived variables previously reported to influence the distribution and percent cover of lichen (Table B in S2 Appendix). For forest stand age, we used forest inventory data provided by forest companies to calculate years since timber harvest, or we used provincial wildfire data to calculate years since wildfire. For climate, we used data from western Canada adjusted for elevation [65] to interpolate climate normals (circa 1961–1991) across our study area. Climatic growing condition data included mean annual precipitation (cm), mean summer precipitation, number of consecutive frost free days, degree-days > 5 °C, and summer heat-moisture index. For forest canopy, in west-central we used a percent canopy cover and height layer derived from LiDAR data [66]. For north-western, we used field-derived visual estimate of canopy cover because LiDAR-derived canopy cover data were not available (S1 Appendix).

For terrain, we used a LiDAR-derived depth to water estimation, a metric of soil wetness based on local topography and modeled hydrologic flow [67,68]. To represent the diminishing effect of the depth to water on vegetation growth, we transformed the variable using an exponential decay function 1 –*e*^−1.55×*Depth*2*Wat*(*m*)^ [69]. This decay function caused depth to water to rapidly decrease at depths greater than 2 m and to become constant at depths greater than 3 m, reflecting the root depth of boreal forest vegetation [70]. We also used the Canadian Digital Elevation Model [71] to extract values of elevation, terrain wetness (compound topographic index, *CTI*; [72]), and solar radiation based on latitude, topographic position, and terrain shadowing intersecting each transect [73,74]. We used CTI rather than depth to water for north-western because LiDAR-derived depth-to-water data were not available for all transects surveyed in that region. We used ArcGIS 10.3 [75] to extract GIS-derived variables intersecting each transect.

### Data analysis

We carried out statistical analysis using R [76] within R-studio [77]–package names are indicated with quotations. To assess differences in mean percent lichen cover among the five sampling strata in each region, we used a Kruskal–Wallis test (‘stat’ [78]) and post hoc pairwise Nemenyi-tests [79] in ‘PMCMR’ [80].

#### Modelling lichen occurrence and abundance

Before analyzing lichen occurrence and abundance, we screened explanatory variables following Zuur et al. [81]. We did not include variables in the same model if they were strongly correlated (|*r*_*p*_| > 0.60); using univariate models and Deviance Information Criterion (DIC, [82]) to identify which of any two correlated variables to include in downstream analyses. We also excluded variables from models with variance inflation factor (VIF) >2 (‘usdm’; [83]). We standardized continuous variables before fitting models (Table C in S2 Appendix).

We used zero-inflated beta regression [84] to model presence-absence and abundance of lichen along transects within each region. Beta regression is appropriate for analysis of proportional data [85–87], and has previously been used to model terrestrial lichen [88]. We fit zero-inflated beta regression models with ‘zoib’ [89,90], which derives inference for model parameters using a Bayesian framework via the Markov Chain Monte Carlo (MCMC) approach implemented in JAGS [91]. We chose a Bayesian framework over a likelihood-based approach because a Bayesian framework helped avoid issues of non-convergence and biased parameters. We surveyed lichen within a 1 m^2^ subplot during the first year of data collection, but increased the subplot size to 10 m^2^ during the second survey year to capture more of the variability present in lichen distribution. We accounted for potential bias in occurrence or percent cover of lichen caused by combining data from 1 m^2^ subplots in the first survey year with 10 m^2^ subplots in the second survey year by including a fixed effect ‘scale’ variable in west-central models (we only surveyed lichen in north-western during 2014). We accounted for the clustered nature of the dataset (i.e., six subplots along a 25-m transect) by treating transect as the sample unit with subplots nested within.

Before fitting models, we combined percent cover of the four lichen genera because combining information from similar rare species improves model predictability relative to individual species models [92]. We built separate models for west-central and north-western, and separate models for *Fire* and *Cut*. We combined *Forest*, *MPB*, and *SingleTree* within a single model. Combining multiple strata into a single model allowed us to explore MPB and single-tree cut-and-burn control effects relative to intact forest by including covariates within the combined model, specifically percent of MPB killed trees and the presence/absence of MPB control along the transect. We expected explanatory variables for occurrence and percent cover to differ, and therefore performed model selection on each part of the zero-inflated equation separately while holding the other side of the equation at the intercept. We evaluated competing models using DIC, and if any two models were within ≤4 ΔDIC of one another, we chose the model with fewer parameters. We considered non-linear effects for the stand age and canopy structure variables by including squared terms (Table B in S2 Appendix). Additional variable details within each strata and region are provided in Table B in S2 Appendix.

We carried out model selection using an iterative process. We started with a global model that included all of the variables of interest for that sampling strata/region (Table B in S2 Appendix), and following the principle of parsimony, we removed uninformative variables [93] from the global model for each sampling strata using a “drop one” approach. For the “drop one” approach, we used DIC to compare alternative models with each variable removed in turn, and removed uninformative variables in an iterative manner from the downstream analysis until removing a variable did not further reduce model DIC. We reported final model results as mean beta (β) coefficients with 2.5% and 97.5% posterior predictive values, or as the relative probability of occurrence (Eq 1) or abundance (Eq 2).

$$1-\frac{e^{\beta Absence}}{1+e^{\beta Absence}}$$ (1)

$$\frac{e^{\beta Abudance}}{1+e^{\beta Abudance}}$$ (2)

Because we modelled presence-absence using a zero-inflated model, positive β coefficients indicate a negative relationship between lichen occurrence and a variable (i.e. probability of lichen being absent), and a positive relationship between percent lichen cover and a variable. We also reported final model results as spatial maps of the predicted mean percent lichen cover given occurrence (Eq 1 * Eq 2) for landscape conditions in 2017. We evaluated the predictive ability of final models using mean absolute error (MAE) and root mean square error (RMSE) calculated from model residuals [94]. MAE is the mean difference between the observed and predicted percent cover in absolute terms. RMSE can be interpreted as the standard error in a model’s unexplained variance. Lower values of MAE and RMSE indicate a better predictive model.

#### Simulating lichen abundance across MPB management actions

To evaluate how percent lichen cover may change in the future under different MPB management approaches, we used the final zero-inflated lichen models to simulate changes. Because the final models for *MPB* and *Control* did not include age since disturbance (see Results), we focused on *Cut* and *Fire*; simulating changes in lichen cover over a forty year period, the maximum age of the strata sample. We used the final lichen model for each strata and region as our baseline landscape condition and evaluated the potential effects of timber harvesting and wildfires on lichen cover into the future by increasing age of the disturbance within models from 0 to 40 years while holding all other variables at their mean. For *Forest*, we added 0 to 40 years to the mean stand age within each region (mean stand age west-central = 119 years, north-western = 97 years). When simulating changes in percent cover of lichen over time, we held all other spatial variables constant at their respective means for each region.

### Model evaluation and caribou habitat selection

To help evaluate the predictive ability of the lichen cover models, we assessed whether caribou in west-central Alberta selected for areas predicted to have higher lichen cover. We focused our analysis on the early and late winter seasons (30 November– 5 February; 6 February– 9 May respectively; see MacNearney et al. [95]) because lichens are an important food resource during winter [39,40]. We used GPS data collected from 100 caribou collared between 1998 and 2016 in the Redrock Prairie Creek population. We only used GPS collar data with a dilution of precision (DOP) ≤ 12 for analysis. We rarefied GPS location data to 2-hr intervals before building models to account for variable fix rates. We then used mixed effects logistic regression within ‘lme4’ [96] to build Resource Selection Function (RSF) models. We constructed RSFs at the home range scale (i.e., 3^rd^ order, [97]); generating 20 random available locations for each GPS location within seasonal caribou home ranges defined by a minimum convex polygon (MCP).

To evaluate the link between caribou habitat selection and lichen, we used a two-step process. First, we used Akaike’s information criterion (AIC) [93,98] and a “drop one” approach to identify a suite of variables related to topography and habitat disturbance to include within a baseline model explaining caribou habitat selection (Table D in S5 Appendix). These variables are known to be important predictors of caribou habitat selection in our study area [95,99,100]. Second, we added the predicted percent lichen cover derived from our zero-inflated lichen models to the baseline model and used AIC to evaluate performance of the baseline model with and without percent lichen cover. We present RSF results as Relative Selection Strength (RSS) and lower and upper 95% confidence intervals (LCL, UCL) of the predictor variables [101]. RSS greater than one indicated a positive relationship between caribou habitat selection and a variable, whereas RSS less than one indicated a negative relationship between habitat selection and a variable.

We evaluated the ability of the final RSF models to predict caribou habitat selection with k-fold cross validation where 20% of the data were withheld for testing [102]. We followed the approach of Boyce et al. [103] and calculated the spearman correlation (*r*_*s*_) between RSF ranked values and the frequency of used points within ten equal area bins across 100 iterations. For k-fold cross validation, *r*_*s*_ values closer to 1 indicate a model with better predictive ability. We also calculated the area under the receiver operator curve (AUC) [104] with ‘caret’ [105]; a measure of model performance [106]. AUC values between 0.7 and 0.8 are considered acceptable discrimination, 0.8 to 0.9 are considered excellent, and above 0.9 is considered outstanding [106].

## Results

### Mean differences in lichen among sampling strata

Mean percent lichen cover differed across strata (west-central χ^2^ = 42.3, df = 4, *P* < 0.001; north-western χ^2^ = 33.0, df = 4, *P* < 0.001; Fig B in S3 Appendix). In west-central, *SingleTree* and *MPB* had lower lichen cover compared to *Cut* (*SingleTree P* = 0.008; *MPB P* < 0.001) and Forest (*SingleTree P* = 0.001; *MPB P* < 0.001). In north-western, *Cut* and *MPB* had lower lichen cover relative to *Fire* (*Cut P* < 0.001; *MPB P* < 0.001) and *Forest* (*Cut P* < 0.001; *MPB P* < 0.001).

### Lichen occurrence and abundance

Final zero-inflated model coefficients are presented in Tables 2 and 3. In west-central, the model for *Cut* indicated that the probability of lichen occurrence increased in conifer forest and was highest at intermediate cutblock age (~25 years; Fig C in S3 Appendix). The *Cut* model indicated that lichen abundance increased with cutblock age. The model for *Fire* indicated that the probability of lichen occurrence increased with wildfire age, but that there was no relationship between wildfire age and lichen abundance. The model for *Forest*, *MPB*, and *SingleTree* suggested that the probability of lichen occurrence was higher in conifer forest, decreased with decreasing stand age, and increased with decreasing summer precipitation. This model also showed that the probability of lichen occurrence was highest when canopy height was ~9 m (Fig C in S3 Appendix), and that percent lichen cover increased linearly with increasing stand canopy height. The model for *Forest*, *MPB*, and *SingleTree* also indicated that the probability of lichen occurrence decreased with increasing percent of mountain pine beetle killed trees.

**Table 2:** Lichen occurrence and abundance model coefficients for the west-central study area.

	Absence			Abundance		
Variable	β	2.5%	97.5%	β	2.5%	97.5%
**Cut**						
Intercept	1.073	0.735	1.423	-2.884	-3.116	-2.658
Lichenscale	-2.219	-2.568	-1.888	-0.553	-0.785	-0.317
CutAge	-1.177	-1.529	-0.821	0.262	0.031	0.516
Cutage^2^	2.798	2.099	3.523	-	-	-
Conifer	-0.958	-1.299	-0.622	0.009	-0.140	0.163
Retention	-	-	-	-0.158	-3.116	0.281
**Fire**						
Intercept	1.600	1.036	2.229	-2.885	-3.269	-2.524
Lichenscale	-0.585	-1.645	0.458	-0.573	-1.285	0.129
FireAge	-2.500	-3.429	-1.638	-	-	-
**Forest|MPB|SingleTree**^a^						
Intercept	7.393	4.908	9.931	-3.098	-3.274	-2.930
Lichenscale	-2.398	-2.618	-2.179	-0.556	-0.732	-0.375
StandAge	-1.092	-1.616	-0.569	-	-	-
Conifer	-0.678	-0.924	-0.436	-0.015	-0.157	0.134
CanopyHGT	-0.539	-0.822	-0.258	0.477	0.297	0.659
CanopyHGT^2^	0.554	0.293	0.821	-	-	-
%MPB	0.387	0.186	0.590	-0.135	-0.318	0.045

Posterior inferences of the coefficients (β, logit scale) of the most parsimonious Bayesian zero-inflated beta distribution model of lichen occurrence and abundance in west-central Alberta, Canada in 2015 across five management or mountain pine beetle scenarios (*Cut*, *Fire*, *Forest*, *MPB*, and *SingleTree*). Mean, 2.5% and 97.5% posterior β predictive values are shown. Variables are explained in S2 Appendix and strata are explained in Table 1.

^a^the SingleTree (ctrl) variable was removed from the final model during model selection.

**Table 3:** Lichen occurrence and abundance model coefficients for the north-western study area.

	Absence			Abundance		
Variable	β	2.5%	97.5%	β	2.5%	97.5%
**Cut**						
Intercept	1.078	0.795	1.372	-3.857	-4.046	-3.671
CutAge	-1.297	-1.824	-0.784	0.320	0.023	0.618
DEM	-1.781	-2.959	-0.715	-	-	-
cti	-1.218	-1.885	-0.548	-	-	-
**Fire**						
Intercept	-0.022	-0.246	0.203	-2.259	-2.582	-1.958
FireAge	-0.419	-0.835	0.000	-	-	-
DEM	0.264	-0.211	0.758	-	-	-
cti	-0.199	-0.656	0.269	-	-	-
**Forest|MPB|SingleTree**^a^						
Intercept	0.538	0.345	0.727	-2.578	-2.875	-2.308
StandAge	-	-	-	0.114	-0.351	0.568
SolarRad	-	-	-	-0.214	-0.638	0.220
%MPB	1.283	0.854	1.726	-	-	-

Posterior inferences of the coefficients (β, logit scale) of the most parsimonious Bayesian zero-inflated beta distribution model of lichen occurrence and abundance in north-western Alberta, Canada in 2015 across five management or mountain pine beetle scenarios (*Cut*, *Fire*, *Forest*, *MPB*, and *SingleTree*). Mean, 2.5% and 97.5% posterior β predictive values are shown. Variables are explained in Table B in S2 Appendix and strata are explained in Table 1.

^a^the SingleTree (ctrl) variable was removed from the final model during model selection.

In north-western, the model for *Cut* indicated that the probability of lichen occurrence increased at dry sites (i.e., high CTI) within cutblocks and at high elevation. This model also showed that lichen occurrence and percent cover increased with cutblock age. The model for *Fire* suggested that the probability of lichen occurrence increased with wildfire age. The model for *Forest*, *MPB*, and *SingleTree* indicated that the probability of lichen occurrence decreased with increasing percent of MPB-killed trees.

**Fig 1:**
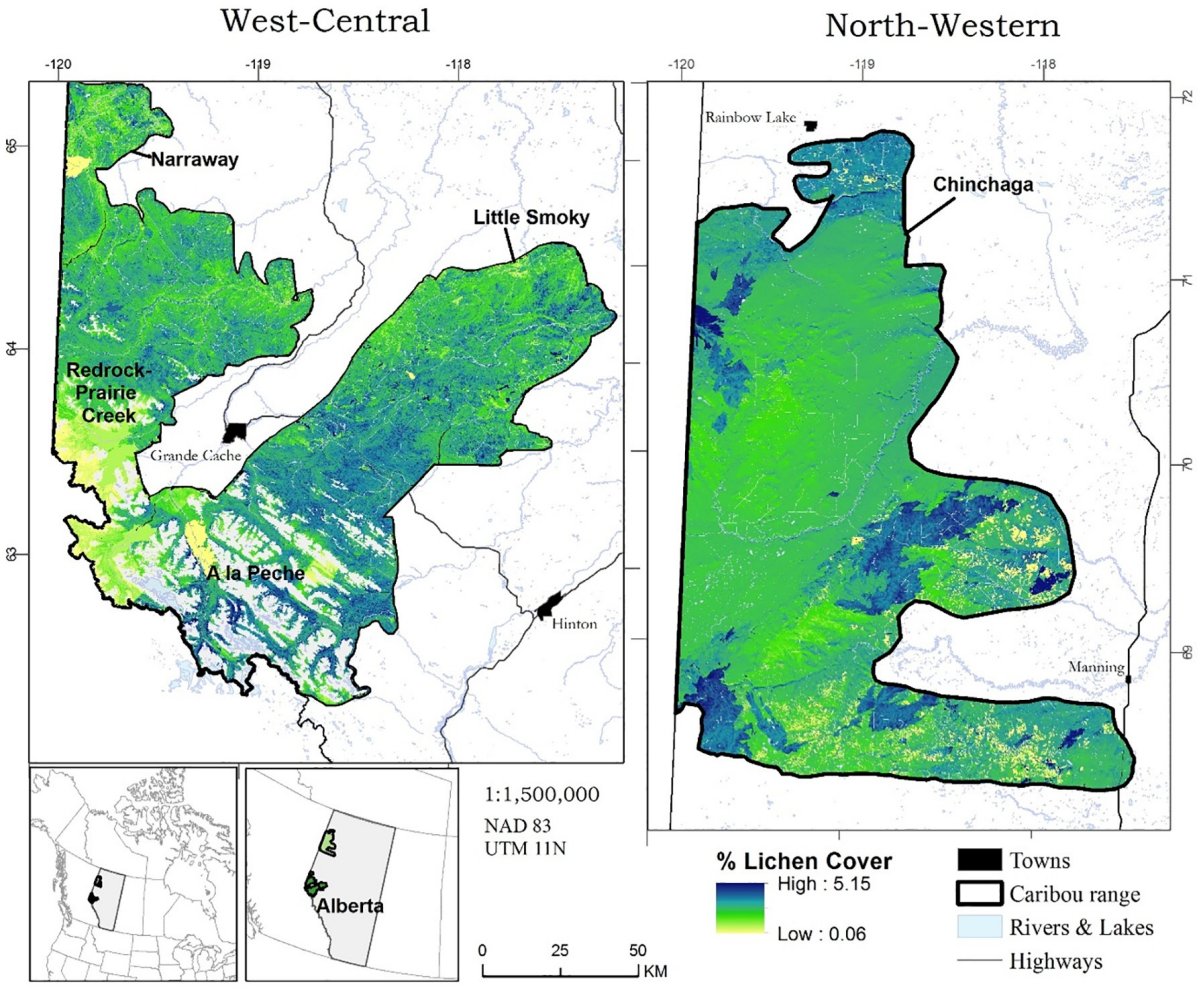
Map of predicted terrestrial lichen cover. Predicted terrestrial lichen cover (*Cetraria* spp., *Cladina* spp., *Cladonia* spp. and *Flavocetraria* spp.) in west-central and north-western Alberta, Canada, mapped using landscape conditions in 2017. Blank areas within caribou range in west-central denote rock and ice covered mountain tops.

In west-central, MAE and RSME pointed to better model fit for the *Forest*, *MPB*, and *SingleTree* model (MAE 1.7%, RSME 1.8%) when compared to *Cut* (MAE 2.7%, RSME 2.9%) and *Fire* models (MAE 1.8%, RSME 1.9%). In north-western, MAE and RSME indicated better model fit for *Cut* (MAE 0.8%, RSME 0.9%), relative to *Forest*, *MPB*, and *SingleTree* (MAE 5.1%, RSME 5.8%) and *Fire* (MAE 9.2%, RSME 10.4%) models. Based on landscape conditions in 2017, we found that in west-central, predicted percent lichen cover tended to be higher in the mountains when compared to the lower elevation foothills (Fig 1; Fig D in S4 Appendix). Higher elevation was associated with older forest stands with lower canopy heights, and with areas with higher mean summer precipitation (Fig E in S4 Appendix).

### Simulating changes in lichen abundance in timber harvested and burned areas

**Fig 2:**
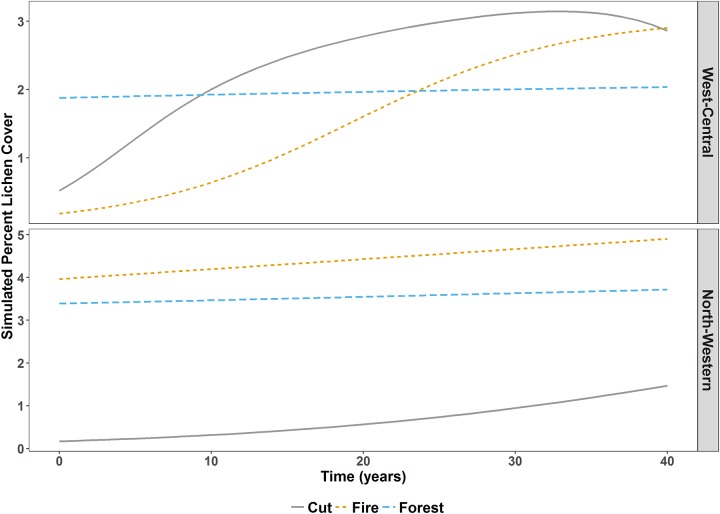
Simulated terrestrial lichen cover under different management actions. Lichen cover simulated over forty years using the zero-inflated lichen models in west-central and north-western Alberta, Canada. *Cut* and *Fire* simulations assumed that the disturbances were created at year zero (2017), whereas for the *Forest* strata simulations the stand age at year zero was the mean stand age in each study area (west-central = 119 years, north-western = 97 years).

In west-central, although *Cut* and *Fire* had the lowest initial percent lichen cover, our models predicted that percent lichen cover in *Cut* and *Fire* would exceed percent lichen cover in *Forest* within forty years (Fig 2). In north-western, *Cut* had the lowest initial percent lichen cover and *Fire* had the highest initial percent lichen cover, and our models predicted that percent lichen cover would remain relatively stable over time (Fig 2).

### Caribou habitat selection and lichen abundance

During early winter, the baseline RSF included elevation, cutblocks, seismic lines, and winter roads, while during late winter, the baseline RSF included cutblocks, seismic lines, and roads. During early and late winter, adding percent lichen cover to the baseline model improved model AIC, fit, and predictive ability (Table 4). The combined base and lichen model indicated that caribou selected areas with higher predicted lichen cover during early (RSS 1.58, LCL 1.57, UCL 1.60) and late winter (RSS 1.63, LCL 1.62, UCL 1.64]. Complete model parameters are in Table E in S5 Appendix.

**Table 4:** RSF model comparison between the baseline and lichen models.

Season	Model	ΔAIC	r_s_ [LCL—UCL]	AUC [LCL—UCL]
Early Winter	Lichen	0	0.95 [0.95–0.96]	0.60 [0.59–0.61]
	Baseline	3942	0.93 [0.92–0.93]	0.58 [0.57–0.58]
Late Winter	Lichen	0	0.99 [0.98–0.99]	0.63 [0.62–0.64]
	Baseline	13387	0.97 [0.97–0.98]	0.54 [0.53–0.55]

Comparison of lichen and baseline RSF models for Redrock-Prairie Creek caribou in west-central Alberta, Canada, between 1998 and 2016, based on Akaike information criterion (AIC), mean spearman correlation coefficient (rs), and area under the receiver operator curve (AUC) with lower (LCL) and upper (UCL) 95% confidence intervals. The rs and AUC were averaged over 100 iterations of a k-fold cross validation, where 20% of the data were withheld for testing.

## Discussion

Landscape management that aims to balance resource extraction and species conservation is complex. Our study evaluated the potential impact of MPB and MPB management on an important winter food resource of threatened caribou. Overall, we found little effect of single-tree MPB cut-and-burn control or standing dead MPB killed trees on terrestrial lichen cover in western Alberta, at least in the eight years after infestation. We did find that timber harvested areas and wildfires had lower lichen cover compared to forests that have not been disturbed, but lichen cover increased with age of timber harvest and wildfire. Our results suggest that in areas where caribou and MPB overlap, single tree cut-and-burn control or leaving infested forest stands as-is may be the preferred management approach. By evaluating the impacts of management actions, the results of this study help to mitigate the unintended consequences of MPB management on caribou where MPB and caribou co-occur.

Our study linking lichen cover to MPB kill and MPB control showed that although lichen abundance was not affected by MPB-killed trees, age of MPB, and MPB control, lichens were less likely to occur in areas with more MPB-killed trees. It is possible that we did not detect an effect of MPB control on lichen cover because cut-and-burn crews operate on foot and during winter, therefore limiting ground disturbances that would damage terrestrial lichen. Even forests timber harvested by mechanized equipment in winter retain high cover of terrestrial lichen, at least in the short term [107]. However, our findings may also be an artifact of the age of the MPB infestation in Alberta because eight years may not be sufficient to detect any appreciable change in slow-growing species such as lichen. Similar research in British Columbia only detected a decrease in lichen cover 10 to 15 years after the initial MPB infestation [108]. It is also possible that the impacts of MPB and MPB control may be more apparent with faster growing understory species like shrubs and forbs [109]. Continuing to assess the availability of lichen in MPB infested and controlled stands at later stages of infestation (> 8 years) would provide additional information to guide forest management decisions.

If we consider alternate MPB management actions, which are timber harvesting and wildfire, our research showed that there was less lichen cover initially in timber harvested and burned areas, but that lichen increased with age. These findings are in line with previous research [110,111], because timber harvested areas generally have more terrestrial lichen cover than fire-origin stands of the same age [112–114]–with low lichen cover after timber harvesting [107]. Lichen cover can remain low in timber harvested stands until they reach 30 years old, with the highest lichen abundance occurring in stands between 50 and 100 years old [113]. Our results and simulations for the north-western region support this pattern of higher lichen cover in burned areas and a slight increase in lichen cover over time. However, in the west-central region, lichen cover in timber harvested areas was slightly higher than lichen cover in burned areas. These regional differences in lichen cover may be driven by differences in local environmental conditions with the north-western region having flat topography and wet conditions relative to the west-central region.

Indeed, even within regions, we found that lichen cover was higher within older, higher elevation, drier mature forests with low-to intermediate canopy heights. The association between lichens, stand age, and stand height was expected because lichens are slow to establish and grow, and they typically reach a peak in abundance within forests with moderate canopy cover and age [115–117]. Forests with more enclosed canopy, humidity, and reduced light transmission to the forest floor are often dominated by mosses [108,113]. We found that lichen cover increased with elevation in the west-central region, consistent with the transition from foothills forests to subalpine areas [58]. In the subalpine, the long 110 to 162 year fire interval [118,119], and harsh climatic conditions [58], likely promote high percent lichen cover by allowing for very old forest stands (i.e., > 300 years), while still maintaining the short-open canopy that lichen thrive in [120]. Our study showed that quantifying relationships between forest attributes and percent lichen cover could help identify forest stands with more abundant winter forage for caribou.

Our habitat selection analysis helped to support the lichen cover models because caribou were more likely to select areas predicted to have greater lichen cover. Other studies that have considered caribou food availability with broad scale habitat characteristics within models have found similar links between caribou and lichen [121–123]. The purpose of the habitat selection analysis herein was to simply evaluate the predictive ability of the lichen cover models. Concluding that lichen cover is the main driver of caribou distribution in west-central Alberta would require a comprehensive comparison of caribou habitat selection relative to lichen cover and competing habitat variables such as terrain, land cover, and predation risk [59,100].

If the ultimate goal of forest management associated with MPB were to retain caribou food supply, then our results would suggest that despite short-term reductions in caribou forage, using timber harvesting and prescribed fire to control MPB could balance management needs and caribou food supply over time. However, we would caution against such an approach because timber harvesting and prescribed fires could have long-term population-level effects on caribou by reducing available caribou habitat [59,123,124], increasing predation risk [45,125,126], and contributing to population declines [36,127]. To address this uncertainty, future studies should expand upon our examination of MPB management actions and caribou food supply by exploring how different MPB management actions change caribou predation risk, especially because unsustainably high predation rates is the primary cause of caribou population declines [128–130].

## Conclusions

We evaluated the potential impacts of managing an invasive forest pest on a species-at-risk. Overall, we found limited evidence that MPB killed trees impact lichen cover. However, our study was restricted to eight years after infestation and management, and further impacts may emerge over time. Leaving MPB killed forest as-is could benefit caribou conservation but this would need to be evaluated against the potential for increased wildfire risk and need to be balanced with socio-economic considerations [131]. Of the four MPB management actions that we evaluated, the MPB cut-and-burn control program appears to balance the need to limit the spread of mountain pine beetle and negative impacts of MPB and MPB management on caribou food. Our work helps address the challenge of managing forests under competing ecological values, specifically species-at-risk conservation versus invasive species control. When developing management strategies across the boreal forest, understanding potential unintended consequences of management actions on non-target species can improve conservation planning in a changing landscape.

## Supporting information

S1 Appendix Details of field data collection among sampling transects. (PDF)

S2 Appendix Explanatory variables used to model lichen occurrence and percent cover. (PDF)

S3 Appendix Relationship between lichen cover and linear variables. (PDF)

S4 Appendix Relationship between predicted lichen cover and linear variables. (PDF)

S5 Appendix Caribou RSF explanatory variables and model parameters. (PDF)
