# Supplementary material for: Caribou in the cross-fire? Considering terrestrial lichen forage in the face of mountain pine beetle (Dendroctonus ponderosae) expansion
Source: PLoS One. 2020 Apr 30;15(4):e0232248. doi: 10.1371/journal.pone.0232248 (PMC7192387; doi:10.1371/journal.pone.0232248)
Supplement: S1 Appendix — (PDF) [file pone.0232248.s001.pdf]

S1 Appendix. Details of field data collection among sampling transects.

Sampling strata

We identified *Cut* strata transects using forest inventory data provided by forest management agreement holders within the study area (West Fraser Timber Co. Ltd, Weyerhaeuser Company, Tolko Industries Ltd., Manning Diversified Forest Products Ltd. [now West Fraser Timber Co. Ltd.], Daishowa-Marubeni International Ltd. [now Mercer International Inc.], and Canfor Corporation). We used historic wildfire perimeter data collected by Alberta Agriculture and Forestry to identify *Fire* strata transects [1]. We partitioned *Cut* and *Fire* strata by time-since-disturbance using increments of five years for *Cut* strata, and increments of ten years for *Fire* strata. We identified *MPB* strata locations using aerial survey data provided by the Government of Alberta; where experienced observers recorded pine trees with red-needles indicative of MPB kill [2]. We also used single-tree cut-and-burn data provided by the Government of Alberta to identify *SingleTree* strata transects [2]. To identify *Forest* transect locations we used a combination of forest inventory data provided by forest management agreement holders and provincial Alberta Vegetation Inventory (AVI) data provided by the Government of Alberta [3].

Field surveys and details of field covariates

Transects were established based on a random location and bearing (Fig A). At each transect, we visually estimated percent cover of lichens within six subplots placed at 5-m increments along a 25-m transect line. In year one in west-central, we estimated percent lichen cover within 1 m^2 subplots, but in year two we increased the subplot size to 10 m^2 to account for clumped lichen growth along transect lines, and the fact that our smaller 1 m^2 subplots often missed patches of lichen growth. In north-western, we estimated percent lichen cover only in 10 m^2 subplots. We chose percent cover as our measure of abundance rather than lichen biomass because recording percent cover reduced survey time, and because percent lichen cover is correlated with lichen biomass [4].

We described forest stand characteristics of each subplot. At each subplot we estimated percent canopy cover visually by standing at the subplot centre and estimate the canopy cover directly above. Along the transect and from the centre of each subplot, we also measured characteristics of the three nearest trees (trees defined as ≥5 cm diameter at 1.3 m height), which we used to estimate the percent of MPB killed trees along each transect, determine whether single-tree cut-and-burn control had occurred, and to classify the transect as non-pine or pine dominant. Field variables are described in Appendix S2: Table B.1.

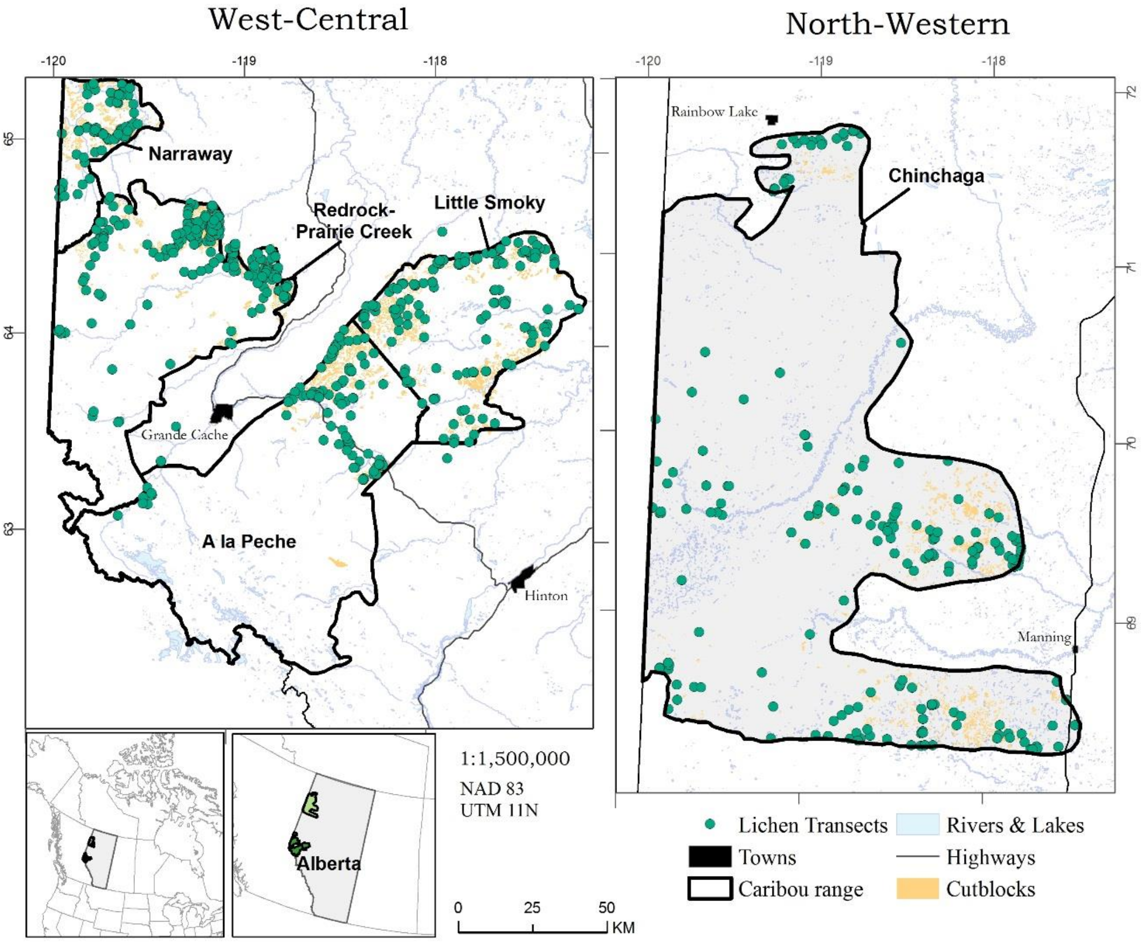

Fig A. Study area in west-central and north-western Alberta, Canada, showing the locations of transects surveyed for terrestrial lichen during the summers of 2014 and 2015.
