# Supplementary material for: Caribou in the cross-fire? Considering terrestrial lichen forage in the face of mountain pine beetle (Dendroctonus ponderosae) expansion
Source: PLoS One. 2020 Apr 30;15(4):e0232248. doi: 10.1371/journal.pone.0232248 (PMC7192387; doi:10.1371/journal.pone.0232248)
Supplement: S2 Appendix — (PDF) [file pone.0232248.s002.pdf]

S2 Appendix. Explanatory variables used to model lichen occurrence and percent cover.

We used GIS-derived variables previously reported to influence lichen occurrence and abundance to model their distribution and percent cover (Table B). These variables included forest age [1–3], climatic growing condition [4,5], forest canopy structure [6–8], terrain [9–11], and intensity of MPB killed trees [12,13].

Table B. Explanatory variables used to model lichen occurrence and percent cover in west-central and north-western Alberta, Canada. Occurrence and percent cover were modeled separately and with different variables given forest disturbance history, specifically regenerating cutblocks (*Cut*), regenerating wildfire (*Fire*), and mature forest potentially with MPB infestation or single-tree cut-and-burn control (*Forest*, *MPB*, and *SingleTree*). An "x" indicates the variable was considered as part of that model.

Variable	Description	Range	Cut	Fire	Forest, MPB, and SingleTree
**Stand Age**					
StandAge	Age of forest stand in years	54 - 404			x
CutAge	Years since timber harvest	1 - 49	x		
FireAge	Years since wildfire	3 - 71		x	
RedAge	Age of MPB red attack	0 - 8			x
CtrlAge	Age of MPB single-tree control	0 - 8			x
**Climate**					
MAP	Mean annual precipitation (mm)	434 - 1008	x	x	x
MSP	Mean summer , from April to September (mm)	271 - 464	x	x	x
FFP	"Frost free period" - number of consecutive frost free days	42 - 92	x	x	x
GrowDD	"Growing degree-days" - Degree-days above 5 °C	402 - 1250	x	x	x
SHM[a]	Summer heat-moisture index (mean warmest month temperature + 10 °C) / (mean summer precipitation (mm). /1000)	21.3 – 57.6	x	x	x
**Terrain**					
Elevation	Elevation (km)	0.46 – 1.97	x	x	x
SolarRadiation	Annual solar radiation energy - watt hours per square meter	739296 - 1253552	x	x	x
CTI	Compound topographic index within a 250 m window	5.3 – 12.2	x	x	x
eDepth2Wat[b]	Depth to water index based on wet area mapping (WAM) from LIDAR data	0 - 1	x	x	x
**Forest Structure**					
ECanopyCov[b]	Visually estimated canopy cover in 10 m^2 subplot	0 - 95	x	x	x
CanopyCov[b,c]	Canopy cover (%) derived from LiDAR	0 - 93	x	x	x
CanopyHGT[b, c]	Canopy height (m) derived from LiDAR	0 - 26	x	x	x
**Retention**					
Retention	Forest retention patch (1), not in retention (0)	0/1	x	x	

**Mountain Pine Beetle**						
%MPB	% Red attack, grey attack, or needle-less tree along the transect.	0 - 100				x
ctrl[c]	MPB single-tree control program (1), not (0)	0/1				x
**Forest Species**						
Pine [c]	Pine dominant trees in subplot (1), not pine dominant (0)	0/1	x	x		
Conifer[c]	Conifer dominant tree in subplot (1), not conifer dominant	0/1	x	x		
LichenScale	Lichen surveyed at 10 m^2 (1), or 1 m^2 (0)	0/1	x	x		x

[a] *SHM* is the ratio between the mean summer temperature and the mean summer precipitation - areas with high *SHM* values are hot and dry during summer

[b] non-linear effects were considered for these variables by including a quadratic term.

[c] only available in the west-central study area due to spatial data limitations.

[d] binary variable.

Table C. Formulas used to standardize the explanatory variables for the lichen occurrence and percent cover zero-inflated models in west-central and north-western Alberta, Canada.

Study Area	Variable	Standardizing Formula
North-western	StandAge	(StandAge - 97.26506)/(2 * 22.7624)
	CutAge	(CutAge - 18.76923)/(2 * 11.44577)
	FireAge	(FireAge - 34.95082)/(2 * 20.9927)
	Elevation	(Elevation - 0.7540574)/(2 * 0.1352632)
	SolarRadiation	(SolarRadiation - 846699.7)/(2 * 24409.17)
	%MPB	(%MPB - 26.33694)/(2 * 36.91969)
	CTI	(CTI - 8.888469)/(2 * 1.070748)
West-central	log_StandAge	log(StandAge)
	CutAge	(CutAge - 20.49223)/(2 * 11.15993)
	FireAge	(FireAge - 12.20833)/(2 * 8.714488)
	CanopyHGT	(log(100 - CanopyHGT) - 4.413199)/(2 * 0.05041127)
	%MPB	(%MPB - 33.56843)/(2 * 37.15046)
	MSP	(MSP - 404.194)/(2 * 31.97229)
