# Supplementary material for: Caribou in the cross-fire? Considering terrestrial lichen forage in the face of mountain pine beetle (Dendroctonus ponderosae) expansion
Source: PLoS One. 2020 Apr 30;15(4):e0232248. doi: 10.1371/journal.pone.0232248 (PMC7192387; doi:10.1371/journal.pone.0232248)
Supplement: S3 Appendix — (PDF) [file pone.0232248.s003.pdf]

S3 Appendix. Relationship between lichen cover and linear variables.

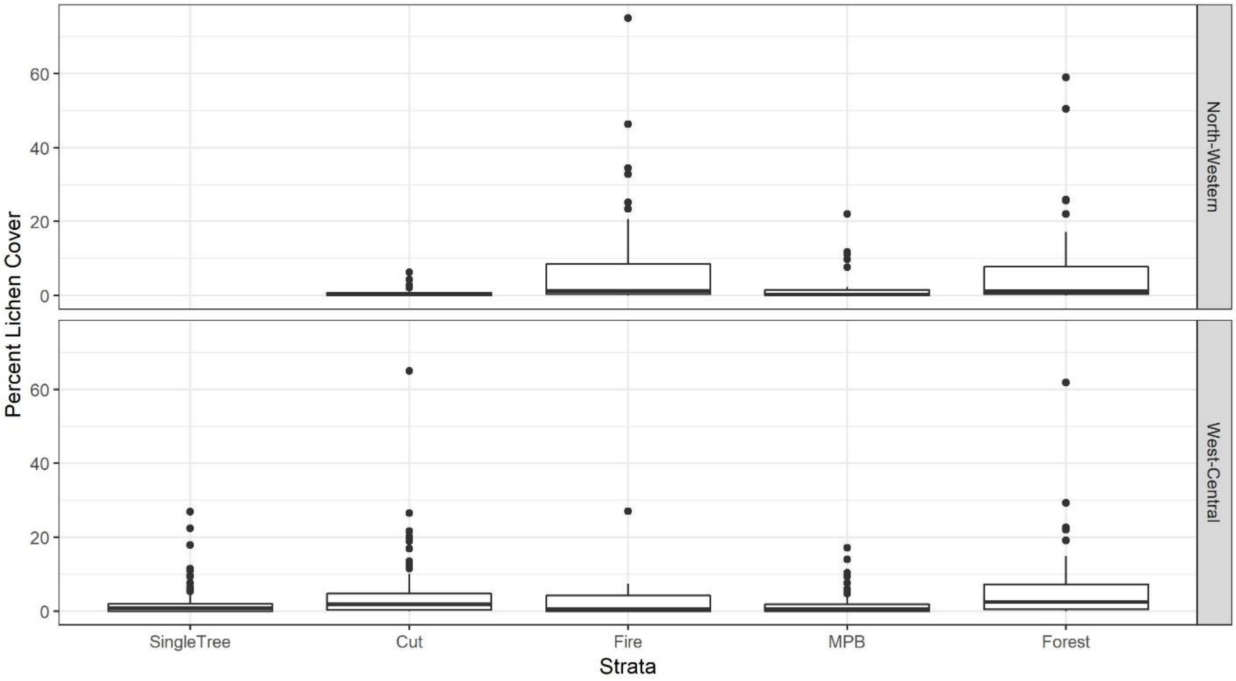

Figure B. Lichen abundance (% cover) by strata. Boxplots showing percent lichen cover among 776 transects surveyed in west-central and north-western Alberta, Canada, during the summers of 2014 and 2015.

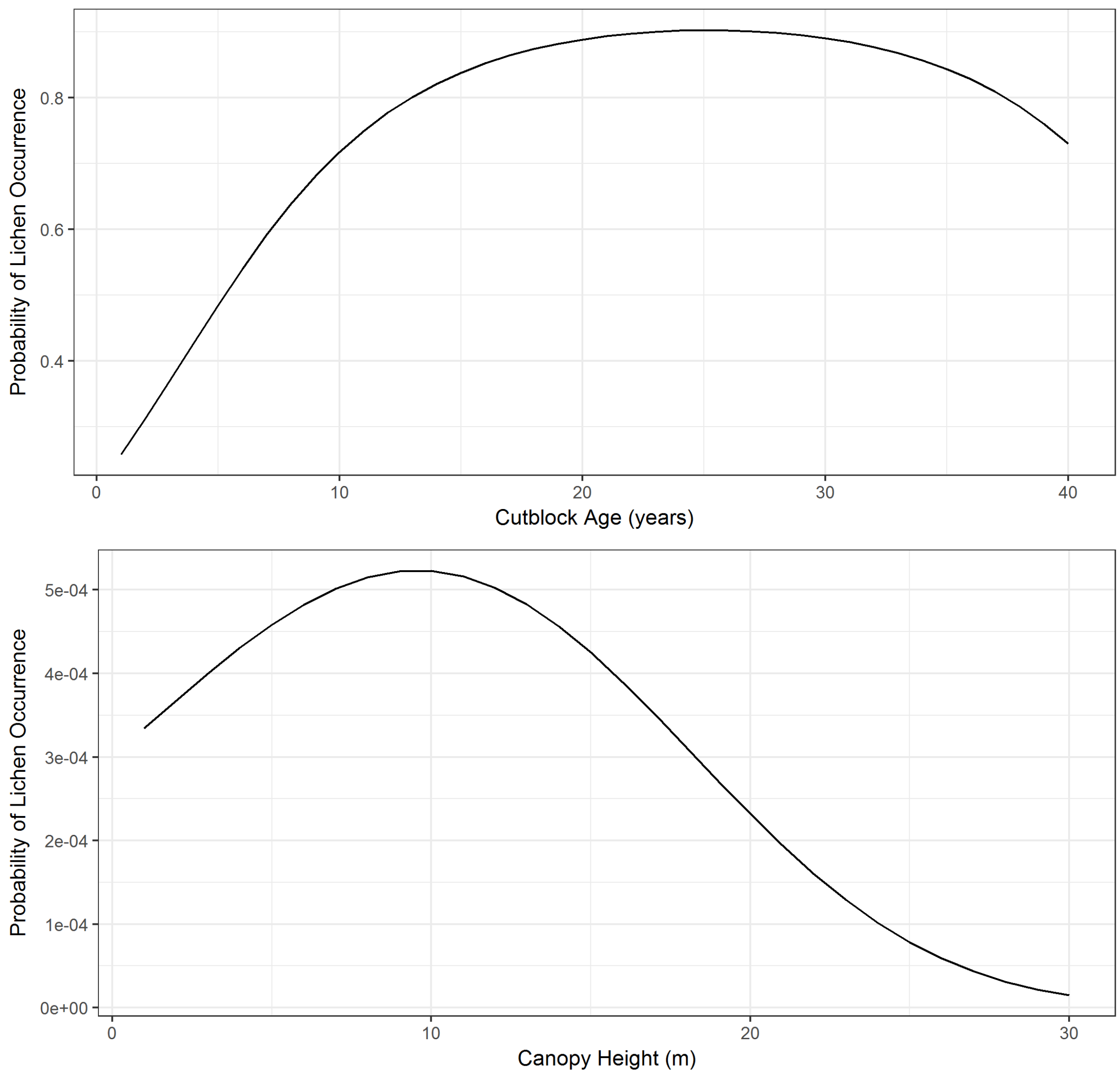

Figure C. Predicted lichen occurrence in relation to cut block age and canopy height in west-central Alberta, Canada. Predicted lichen occurrence in relation to non-linear squared variables - cut block age (*Cut* strata) and canopy height (combined *Forest*/*MPB*/*SingleTree* strata), based on the zero-inflated lichen model for west-central Alberta, Canada, in 2015. Model coefficients are in Table 2.
