# Supplementary material for: Caribou in the cross-fire? Considering terrestrial lichen forage in the face of mountain pine beetle (Dendroctonus ponderosae) expansion
Source: PLoS One. 2020 Apr 30;15(4):e0232248. doi: 10.1371/journal.pone.0232248 (PMC7192387; doi:10.1371/journal.pone.0232248)
Supplement: S4 Appendix — (PDF) [file pone.0232248.s004.pdf]

S4 Appendix. Relationship between predicted lichen cover and linear variables.

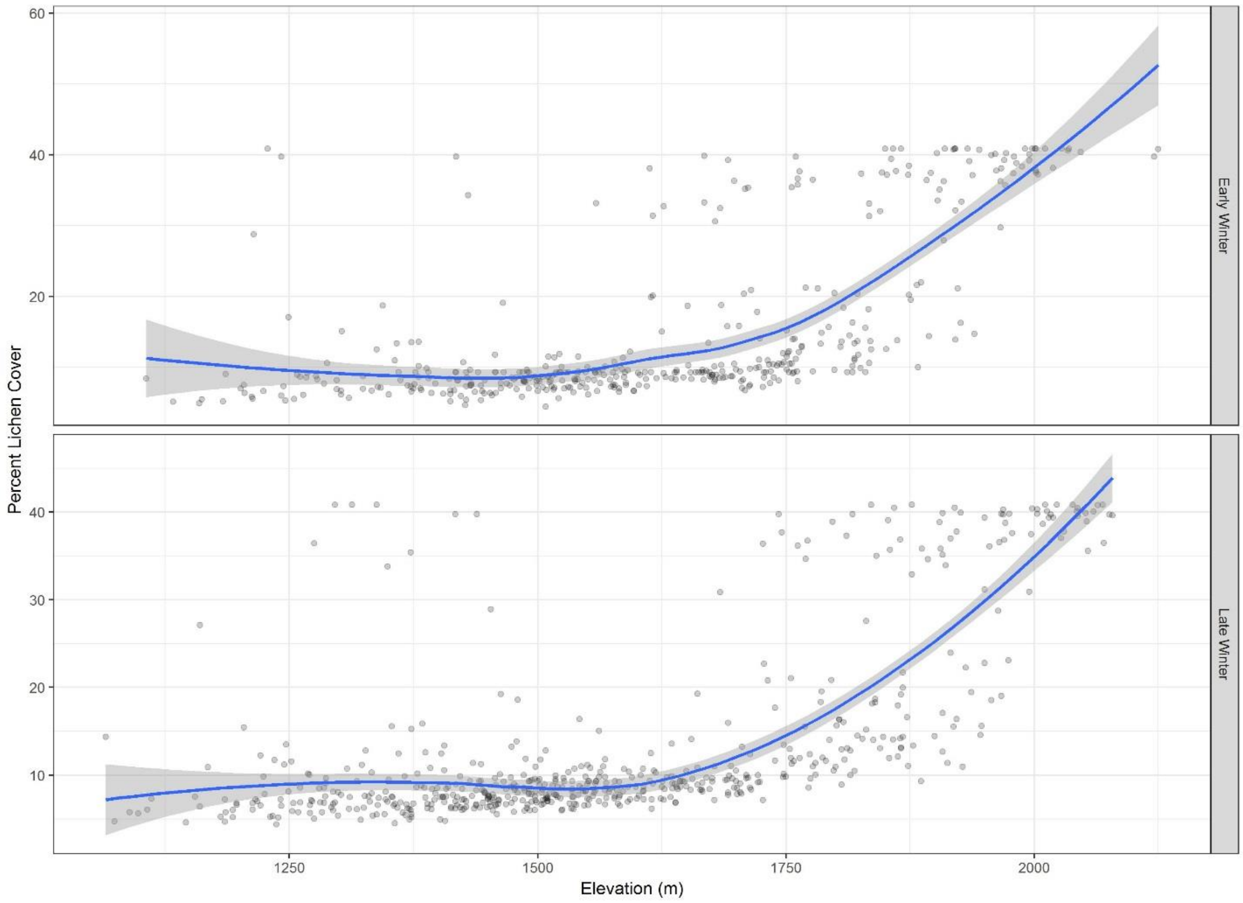

Figure D. Lichen and Elevation Comparison. Comparing elevation (m) to predicted percent lichen cover in north-western and west-central Alberta during early and late winter seasons (30 November – 5 February; 6 February – 9 May respectively; see MacNearney et al. [1] for details on how early and late winter seasons were derived). Shown is the loess smooth fitted curve and shaded areas are the standard error.

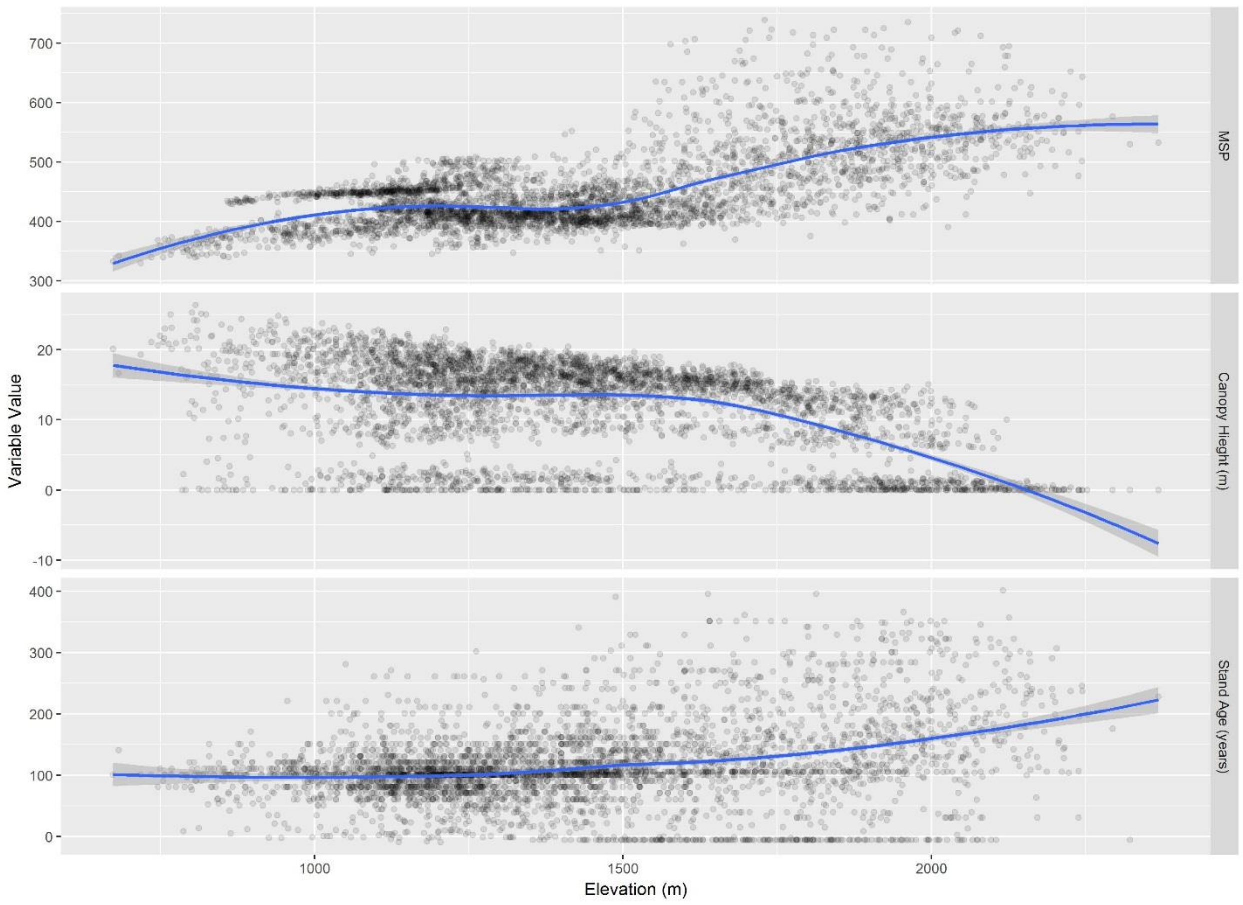

Figure E. Comparing elevation with other variables. Comparing elevation (m) to variables within the zero-inflated lichen model for mature forest that could explain the relationship between predicted lichen cover and elevation. Shown is the loess smooth fitted curve and shaded areas are the standard error.
