# Supplementary material for: Caribou in the cross-fire? Considering terrestrial lichen forage in the face of mountain pine beetle (Dendroctonus ponderosae) expansion
Source: PLoS One. 2020 Apr 30;15(4):e0232248. doi: 10.1371/journal.pone.0232248 (PMC7192387; doi:10.1371/journal.pone.0232248)
Supplement: S5 Appendix — (PDF) [file pone.0232248.s005.pdf]

S5 Appendix. Caribou RSF explanatory variables and model parameters.

Table D. Explanatory variables used to model caribou habitat selection in west-central and north-western Alberta, Canada between 1998 and 2016. Coefficients for the zero-inflated models are in Tables 2 and 3.

Variable	Description	Range
**Anthropogenic**		
$Seismic_{1k}$[a]	Density (km^2/km^2) of seismic lines within 1 km circular radius	0.00 – 0.05
$Cutblock_{1k}$	Density (km^2/km^2) of cutblocks within a 1 km circular radius	0.00 – 0.92
$Road_{1k}$[a]	Density (km^2/km^2) of roads within 1 km circular radius	0.00 – 0.13
**Terrain**		
Elevation	Elevation (m)	905 – 2360
**Lichen**		
%Lichen	Percent lichen cover predicted with zero-inflated models	2.39 – 40.84

[a] linear features were buffered by 30 meters to calculate density.

Table E. Relative Selection Strength (RSS) and associated lower (LCL) and upper (UCL) 95% confidence intervals for caribou resource selection function (RSF) models among the Redrock-Prairie Creek herd in west-central Alberta, Canada.

Season	Variable	RSS [LCL, UCL]
Early Winter	Cut1k	0.937 [0.927, 0.947]
	Lichen	1.584 [1.572, 1.596]
	Seis1k	1.223 [1.207, 1.240]
Late Winter	Road1k	0.923 [0.915, 0.930]
	Cut1k	0.969 [0.960, 0.977]
	Lichen	1.631 [1.620, 1.643]
	Seis1k	1.522 [1.505, 1.539]
